# Midwives’ views on factors that contribute to health care inequalities among immigrants in Sweden: a qualitative study

**DOI:** 10.1186/1475-9276-11-47

**Published:** 2012-08-18

**Authors:** Sharareh Akhavan

**Affiliations:** 1 Department of Public Health - University of Skövde & School of Health, Care and Social Welfare, University of Mälardalen, Mälardalen, Sweden

**Keywords:** Immigrants, Midwives, Communication, Inequality, Transcultural health care

## Abstract

**Introduction:**

Ethnic and socioeconomic inequalities in the Swedish health care system have increased. Most indicators suggest that immigrants have significantly poorer health than native Swedes. The purpose of this study was to explore the views of midwives on the factors that contribute to health care inequality among immigrants.

**Methods:**

Data were collected via semi-structured interviews with ten midwives. These were transcribed and related categories identified through content analysis.

**Results:**

The interview data were divided into three main categories and seven subcategories. The category “Communication” was divided into subcategories “The meeting”, “Cultural diversity and language barriers” and “Trust and confidence”. The category “Potential barriers to the use of health care services” contained two subcategories, “Seeking health care” and “Receiving equal treatment”. Finally, the category “Transcultural health care” had subcategories “Education on transcultural health care” and “The concept”.

**Conclusions:**

This study suggests that midwives believe that health care inequality among immigrants can be the result of miscommunication which may arise due to a shortage of meeting time, language barriers, different systems of cultural beliefs and practices and limited patient-caregiver trust. Midwives emphasized that education level, country of origin and length of stay in Sweden play a role when an immigrant seeks health care. Immigrants face more difficulties when seeking health care and in receiving adequate levels of care. However, different views among the midwives were also observed. Some midwives were sensitive to individual and intra-group differences, while some others viewed immigrants as a group of “others”. Midwives’ beliefs about subgroup-specific health services vs. integrating immigrants’ health care into mainstream health care services should be investigated further. Patients’ perspective should also be considered.

## Introduction

The practice of health care in Sweden has encountered new challenges in recent decades as the immigrant population has increased. The goal of the Swedish health care system is to provide good care on equal terms to all people and in so doing, contribute to a more equitable spread of health [1]. Health care in Sweden is a public responsibility, financed primarily through taxes that are levied by county councils and municipalities. The Swedish health care system is structured on three levels: national, represented by central government, regional, i.e., the municipalities and local, represented by the county councils. The county councils plan the development and organization of health care according to the needs of their residents, among others immigrants. However, asylum seekers and undocumented immigrants in Sweden have very restricted access to state subsidized health care [2,3].

Reports show that inequalities in the Swedish health care system have increased since the beginning of the 1990s. Most indicators suggest that immigrants have significantly poorer health than native Swedes [4,5]. Although the increasing disparity may have different causes, one may be due to the fact that immigrants do not seek health care when they need to and do not receive the treatment that they need when they seek care [4]. This study is one segment of a large study which has been conducted to explore factors that contribute to inequalities in the provision of health care in Sweden. Midwives were chosen as the study group because the perinatal period is often the first contact that a newly arrived immigrant family has with the health care system, and that experience will affect future use of the system [6]. Furthermore, midwives are responsible for a high percentage of obstetrics care in Sweden [7]. As such, midwives play a crucial role as the representatives of the larger health care system for immigrants.

Today, roughly 20 percent of the Swedish population are immigrants or descendants of immigrants, i.e., they were either born outside of Sweden or have at least one parent who was born outside of Sweden [8]. The term “immigrants” will therefore be used to refer to both groups throughout this paper. It cannot be ignored, however, that the term ‘immigrants’ encompasses a very diverse group comprising people from different countries and with different socioeconomic backgrounds. Over the years there have been various patterns of migration to Sweden. During the 1950s and 1960s, labor migration resulted in an increased number of immigrants from countries such as Italy, Greece and Turkey. During the 1970s and 1980s, war and the political situation in countries such as Chile, Iran and Iraq resulted in refugees entering Sweden. The last two decades have been characterized by migration from countries such as Yugoslavia and Somalia, where civil war has threatened the life and health of people [9]. Most immigrants will primarily be from European countries outside of the European Union, Africa, Asia and Latin America [8]. What these people have in common is the experience of ethnic discrimination [10].

Immigrants in Sweden experience worse physical and psychological health compared with native Swedes [4,5]. There are differences in healthcare utilization. The Statistics Central Board’s [4] study showed that 21 percent of immigrant women reported need of health care but had not sought it (self-reported), in comparison with 12 percent of native Swedish women. The study [4] showed that the rate of preventable mortality (death due to illnesses that the health care sector is equipped to address through the application of preventative or targeted medical treatment) is higher among immigrants. Immigrants are treated unequally within the Swedish health care sector; the use of well-documented medical treatments, for example for heart attack, heart failure, stroke and chronic obstructive pulmonary disease is lower among immigrants than among native Swedes [11].

The factors that contribute to health inequality due to immigrant status and cultural differences are complex and varied. Lack of available information, communication difficulties [12] and lower levels of trust in the health care system [9,13] are some factors that have been discussed. Ethnic discrimination [14,15] and insufficient clinical follow-up treatments and/or fewer post-operative checkups [16] are other factors that have been mentioned in earlier research.

The aim of this study is to explore the views of one group of health care professionals (midwives) on the factors that contribute to health care inequality among immigrants.

## Methods

A qualitative approach was chosen to obtain a deeper understanding of the midwives' views on inequalities in the provision of health care due to immigrant status and cultural differences. Based on the objective, semi-structured interviews were considered to be the best method, with all interviewees being asked the same questions. The use of semi-structured interviews enables the researcher to prepare a number of questions in advance. The interviewer may also ask spontaneous questions and change the order of the set questions as the interview progresses. Semi-structured interviews also allow the interviewees to recount their experiences with as little guidance as possible from the interviewer [17]. The questions were open-response alternatives, creating equal opportunities for all midwives to share their views and experiences [18].

### Participants

The midwives or the superintendent of units in two municipalities in a city in western Sweden were informed about the study by telephone or via e-mail and appointments were made with those who were interested in being interviewed. The municipalities were selected randomly from a group of 20 that had a higher number of immigrants. The municipalities with a higher number of immigrants were identified from the segregation index that was calculated for all municipalities in Sweden for the years 1997–2006 [19]. The criteria for being included in the study were that the midwives were professionally trained and had worked in the selected district for at least 12 months. Ten midwives, all native Swedes, were interviewed. Their mean age was 49.2 years, with a range of 35–57. Most of them had between 6–25 years of experience in the field and worked often, or almost always, with immigrant women (Table 1). 

**Table 1:** The interviewed midwives

**Participants**	**Age**	**Workplace**	**Education**	**Number of years in the profession**	**Reported frequency of working with immigrant patients**
1	55	Municipality 1	HealthCare College in Gothenburg (HCCG)	15	Often
2	45	Municipality 1	HCCG	8	Often
3	45	Municipality 2	Health Care College in Stockholm (HCCS)	9	Often
4	47	Municipality 2	HCCG	13	Often
5	55	Municipality 1	HCCG	18	Almost always
6	44	Municipality 2	HCCG	12	Almost always
7	55	Municipality 2	HCCG	14	Almost always
8	54	Municipality 1	HCCG	18	Almost always
9	57	Municipality 1	HCCG	25	Almost always
10	35	Municipality 2	HCCS	6	Often

### Data collection

Each midwife was interviewed individually and in a quiet environment that the midwife selected. The interviews lasted between 50-60 minutes. Audio recordings were made of all interviews. The interviews were conducted between January 2009 and February 2010. The interviews were transcribed and translated from Swedish to English by the author and a research assistant. The questions posed were open-ended in order to obtain spontaneous information on the study’s purpose. The research questions were prepared as Lofland & Lofland [20] suggested, by considering ‘Precisely what about this thing is puzzling me?’ They suggested that the puzzlement can be stimulated by various activities, such as discussions with colleagues and studying existing literature on the topic. The research questions were: What happens during the meeting with an immigrant woman? What are your opinions on inequality in health care? How can inequality arise in the meeting with an immigrant woman? What are your thoughts on transcultural health care?

A Research Assistant with a Master’s degree in Public Health assisted in preparing the research questions, as well as with conducting and analyzing the interviews. This was to ensure that the analysis was conducted by two individuals with diverse professional backgrounds, in order to balancing existing individual biases.

The basic requirements of this study were that oral and written information be provided to participants and that written consent be obtained from them. The interviews were voluntary and informants were able to terminate the interview without justification. Privacy issues were considered when noting the midwives' names. Participants will therefore remain anonymous. The study was approved by the Ethical Committee in Gothenburg (Dnr: 262–09).

### Data analysis

A qualitative content analysis method [17] was used to analyze the midwives' views. Each interview was printed on paper and read through several times before and during the analytical process by the author and her research assistant, independently of each other. This was in order to check that their interpretations were similar. The first step in the analytical process was to pick up meaning-bearing units, each related to the purpose of the study. A meaning-bearing unit is a paragraph or sentence that highlights the content of the material (Ibid). The next step was to shorten the chosen meaning-bearing units to condensed units, i.e., to make the content more manageable but still maintain the parts that were considered to be of importance. The next step in the analytical process was to pick codes out of the condensed units. This was done to flag the contents for a higher level of analysis and to briefly describe the contents. The codes may be, as Granheim & Lundman [21] described them, discrete objects or phenomena that are related to the context. The author and her research assistant agreed upon the codes and the created subcategories and categories before proceeding. The criteria for inclusion of a coding category were (1) how relevant the codes were to current study’s aim and (2) whether the code actually emerged in the text. Categories were initially kept as broad as possible without overlapping. Therefore few categories are chosen in the initial stages of the analysis. Then, as more data accumulated, the major categories were sorted into three categories [22-24]. These three categories were compared with the entire body of interviews in order to verify their original contexts. Furthermore, two external co-analyzers read the transcribed interviews and drew conclusions regarding the main content of each interview. Their findings were discussed with the author and their conclusions regarding the contents of the interviews agreed well with the authors’ coding. Finally, the analytical consistency was investigated by the author (Table 2). 

**Table 2:** Examples of meaning units, condensed meaning units and codes

**Meaning unit**	**Condensed meaning unit**	**Code**	**Subcategory**	**Category**
It is important to let the immigrant woman herself say what she needs and that the midwives then follow up on these needs and try to make the meeting a positive experience	Important to let the immigrant woman herself say what she needs	Listen, follow up and make the meeting a positive experience	The meeting	Communication
	midwives then follow up on these needs			
	to make the meeting a positive experience			

## Results

The interview data were divided into three main categories and seven subcategories. The first category “Communication” had three subcategories, “The meeting”, “Cultural diversity and language barriers” and “Trust and confidence”. The second category “Potential barriers to the use of health services” had two subcategories, “Seeking health care” and “Receiving equal treatment”. Finally, the third category “Transcultural health care” had two subcategories, “Education on transcultural health care” and “The concept”.

### Communication

The results from all the interviews showed that communication has a central and significant role and may contribute to health inequality owing to ethnic and cultural differences.

#### The meeting

According to the midwives there was a need for an "open" and welcoming meeting. By “open” they meant that it was necessary to listen and to consider the needs of immigrant women. *“It is important to let the immigrant woman herself say what she needs and that the midwives then follow up on these needs and try to make the meeting a positive experience”.*

Time was another aspect that had to be considered. The need for an advanced consultation might arise during the meeting, but the time allotted for a meeting was very limited and midwives were unable to extend the time available. The results showed that the midwives experienced inadequate time as a factor that might contribute to inequalities in healthcare. *“A meeting with an immigrant woman demands more time; for example, more time to explain and to get confirmation that she understands. We have a set time for each patient and this cannot be extended”.* Another midwife argued *"It's obvious that everyone should get good care, but time limitations may restrict the provision of good care on equal terms. For example, we need a longer period of time when we use an interpreter. It's very important that we understand each other“.*

#### Cultural diversity and language barriers

According to some of the midwives language was an essential instrument for promoting effective communication. Good language skills could reduce inequalities in the provision of health care. *"There may be language problems … It’s important to use professionally trained medical interpreters".* It was not always feasible to use an interpreter. One midwife stated “*It would be much better if the patient could speak Swedish".* She added *"Sometimes even with an interpreter it becomes difficult to understand, because, we naturally use a great many difficult words in health care".* For some immigrant groups which came from countries with ethnical diversity and different languages and accents, the choice of interpreter was important. One midwife said that *“the interpreters’ accents and ethnic identities can sometimes be problematic”.* Other midwives said language should not be regarded as a factor that contributes to health care inequality. One midwife said *“It does not matter what a woman's cultural background is or what her skills in the Swedish language are … As a midwife I should provide good care”.* Another one added *“midwives should adapt their way of communicating”.*

Cultural differences and the response of health care staff to these differences were mentioned as another factor that may lead to inequalities in health care provision. Differences in cultural beliefs, behaviors and expectations may lead to misunderstanding and miscommunication. Some midwives mentioned that there were some cultural collisions between immigrants and health care staff because of the patriarchal culture or religious beliefs, etc. One said *“These kinds of beliefs can affect the immigrant men and women when making decisions, for example about abortion … we can only inform, we cannot contribute in any other way”.* It was important to give the immigrant woman the feeling that she could choose, that she had control and that it was her decision. One midwife gave an example: *“Different women from different cultures give birth in different positions and we try to understand and adapt … no way is wrong … the aim is to deliver a healthy baby and that the mother feels good”.*

#### Trust and confidence

The midwives all agreed that it takes time to establish trust and confidence in a meeting. In order to provide good care on equal terms, it was *"important to understand and to trust in each other".* According to the interviewed midwives, some policies might damage the establishment of trust and confidence between the care provider and the patient. An example of this was the Swedish health care guidelines to X-ray pregnant women from certain countries because of the risk of tuberculosis. One midwife said *“normally pregnant women should not be X-rayed, but in the case of immigrant women there is an exception and some immigrant women refuse to do it because of the pregnancy and they mistrust the system that has this policy”.* Mistrust might also develop due to a lack of medical knowledge and language barriers. A midwife gave this example: *“It is difficult to give information about fetal diagnosis through an interpreter and talk about probability here and probability there. These difficulties in communication can establish mistrust”.* Another midwife believed that trust could be established by allowing immigrant women to disclose their medical histories without fear of immigration authorities.

“For example, Somalian women may have children that are not their own, they just raise them as their children to save their lives, but for me as a midwife is important to know how many children she has given birth to. If I can show that I am a health care staff and that I have no contact with the immigration authorities and if I give her a chance to narrate her history, listen and show understanding, then she will trust me”.

### Potential barriers to the use of health care services

The interviewed midwives believed that inequality in health care could be more easily identified by investigating health-seeking behavior and received treatment.

#### Seeking health care

The majority of the midwives observed no major differences in the seeking of health care between immigrant and native Swedish women. However, a few midwives had another view. One said *“some immigrant women are used to difficult conditions and seek health care when it may be too late".* Generally, based on their experiences, midwives felt that a woman's level of education, country of origin and length of stay in Sweden could affect her views on how she uses the health care services. The midwives regarded level of education as a more important factor than cultural differences. One said *“Education is more important than culture, the more educated (the woman is), the fewer the differences, but the woman is still shaped by her culture".* Another midwife remarked: *“Just because you are immigrants it doesn’t mean that your health care seeking behavior differs so much from that of native Swedes”.* She continued, *“some women are isolated, do not speak Swedish and have no contact with the Swedish society. They are newly arrived or have been here for a short time… for them, seeking health care when they need it is a problem, especially when they have serious problems like high blood pressure during pregnancy”.*

#### Receiving equal treatment

The midwives all agreed that immigrant women’s status could affect how they are treated in the health care system. Furthermore, they assumed that immigrant women did not receive the same treatment and care as native Swedish women. One midwife gave an example: *“newly arrived immigrant women may not have interpreters during the birthing process. This is terrible and can create lots of problems for care givers and for mothers”.* Another said *"… I think there are big differences for those from other countries regarding how they are treated and how treatment works …perhaps due to ignorance or prejudices".* Another one added *"I can imagine that a Swedish couple who is highly educated receives very different care and treatment in a hospital than a couple from a different culture who does not speak any Swedish".* The reason that people are treated differently in the health care sector, according to the midwives, is that immigrants cannot demand their rights. One midwife said *“It is perhaps that it is hard to assert their rights for health care. One has to express oneself well. And in many cases, immigrants are not as good at it as the Swedes".* Another midwife mentioned that *"The vulnerable groups in society have more difficulties in getting adequate care … I believe that many people who come from other countries unfortunately count as a vulnerable group".* One of the midwives mentioned that immigrants and native Swedes do not get the same care *“because those born abroad have more difficulties in making their voices heard in the health care sector”.*

There were two different ways of thinking about receiving equal treatment. Some midwives believed that it was the responsibility of the society and the health care services to be able to provide equal treatment to all citizens. One said *"They need a better introduction to the society so that they know how it works. They should also get the opportunity to learn Swedish and to acquire good language skills so they can get better care”.* According to one midwife *“It is very important that society takes responsibility and provides information. If you know what rights you have, and above all, have knowledge of what health care can help with … immigrants do not know what they can get help with".* And another midwife said *“It’s our responsibility to have better knowledge of different cultures in order to improve their chances for receiving equal treatment".* One midwife, however, expressed her confusion: *“I don’t know if it depends on attitudes and prejudices in the Swedish health care system or on immigrants’ lack of knowledge of how the system works”.* Indeed, some midwives believed that there could be differences in treatment and access to care, but *"… It is not always the health care services that are the problem"*. They believed that is an individual’s (the immigrant patient’s) responsibility to know the system, to speak the language and be able to express herself. *“An individual's ability to express herself and understand is critical to the standard of received treatment in the health care system".*

### Transcultural health

#### Education on transcultural health care

All midwives expressed the opinion that there should be more on the subject of transcultural health care in their education and training in order to improve their communication skills and enable them to provide equal and good health care. The midwives said that they needed continuous training in cultural diversity. One said *“The world is constantly changing and people are moving to Sweden for various reasons. Midwives would like to continuously update their knowledge of different cultures”.* One said "*In the 1990s we had a lot of information, especially when large groups came from Somalia. But now it's like you have to seek the information yourself".* During their training they had no courses on cultural diversity or cultural sensitivity. Training in transcultural health care meant different things to different midwives. One midwife said *“one cannot learn about all different cultures … cultural sensitivity training means to learn to accept, respect and be keen and open”.* Another believed that health care staff needed training in ethnic and Eurocentric attitudes. ”*I wish that we could learn about ethnicity and culture during our training… I want to learn how to meet culturally diverse people in the right way …we have to improve our ways of communication and our cultural competency”.*

#### The concept

“Transcultural health care” was an unfamiliar expression to most of the midwives who were interviewed in the study. One said *"not words we use, but we are caring in a cultural way, it means trying to be observant and trying to capture what is different".* One added *"I have never heard the term but I think different cultures have different beliefs and that is the only difference”.*

Although the expression was unfamiliar, the midwives had ideas about the concept of transcultural health care. One said *“we live in a society which is culturally diverse and the health services should be more aware that people come from different cultures. It is something that must be accepted and respected”.* To work transculturally, according to one midwife, meant *“to adapt our knowledge and experience to different cultures”.* Another said *“transcultural health care means to work beyond the borders and norms”.* Some midwives believed that transcultural health care was about *"cultural communication”* and viewed immigrants as a group. One midwife said *“I try to see and understand how they express themselves".* Another explained *"… we should learn how people from other cultures act … society must also have an understanding of it. We come from different cultures and it has to be respected in order for everyone to feel welcome".* The fact that immigrants were viewed as a homogenous group was emphasized by another midwife *“For me it is like going abroad, I try to place myself in their culture and their world and to think with their brains …".* Some midwives had a different view of transcultural health care; for them it was mostly about seeing the individual. One said *“I try to see who I have in front of me and form my idea of what she reflects and expresses. I am not programmed to run the same procedure for everyone”.*

All midwives agreed that having culturally diverse health care staff was an important resource for providing culturally sensitive health care, but they were all negative about the idea of ethnic health care services. One said *“Then we will have even more segregation* “. And another added “*I think we can learn from each other. The native Swedish health care staff can learn from staff who are immigrants and vice versa. Employing immigrant health care staff will facilitate this”.* According to the midwives, another negative aspect of ethnic health care services would be that they would provide low quality care because they would get fewer resources and qualified health care staff would not work in such services. One midwife said of such a health care service: *“Nothing will work, staff will leave, we must make it attractive to work with culturally diverse patients and not establish segregated health care services”.* Another midwife stated that establishing ethnic health care services *“will cement prejudices”.*

## Discussion

The findings of this study show that midwives view communication as having a central role that may contribute to health inequalities. An open meeting in which the care provider (in this case the midwives participating in the study) allows for adequate time to listen to and consider the needs of the patient and a meeting in which the cultural and language differences do not lead to misunderstandings are factors that contribute to the provision of equitable health care. Midwives believe that the potential barriers to the use of health care services are immigrants’ health care seeking behavior and the way immigrants are treated in the health care system. Finally, the questions on transcultural health care shed light on two different perspectives on immigrant patients; they are either viewed as (a) individuals or (b) a group. Furthermore, all midwives agreed that having culturally diverse health care staff was an important resource for providing culturally sensitive health care, but they all responded negatively to the idea of ethnic health care services.

### Communication

The results of the interviews show that midwives believe that poor verbal communication or language skills may lead to miscommunication which in turn may contribute to inequalities in the provision of health care. In agreement with the results of this study, previous research articles [25,26] mention the quality of verbal communication and language skills as factors that may contribute to inequality in health care. Fortier et al. [27] assert that a failure to ensure adequate communication between patient and provider “can lead to inappropriate or unnecessary testing, clinical inefficiency, misdiagnosis, negative outcomes and malpractice.”

Previous research [28,29] indicates that language barriers can adversely affect the quality of care. Some researchers point out that when a patient does not speak the language of his or her health care provider, multiple adverse effects on the patient’s health may occur and lead to poor patient satisfaction, poor compliance and underuse of services [30,31]. Some interviewed midwives emphasized that as caregivers they should provide good care, regardless of whether their patient can speak the language or not. In other words, language should not be a barrier to providing equitable health care. Employing bilingual health care staff, using qualified interpreters or using community-based health navigators (CBHN) [32] and providing written information in different languages may facilitate communication, increase patient satisfaction and increase patient understanding. It would also help to avoid errors in diagnosis and treatment and avoid the costs of employing telephone interpreters [33,34]. Almost all communication between midwives and immigrant patients was conducted through an interpreter, which meant that it took longer to communicate all of the information. The use of an interpreter could not be avoided; this was a tool that the midwives felt that they had to work with in order to provide good care on equal terms. According to the midwives, using professionally trained medical interpreters can provide a higher degree of accuracy and confidentiality and increased overall effectiveness. However, even this approach is not without potential problems. For example, the information advantage is lost when health professionals are not aware of how much information was translated by the interpreter [35] or when the interpreter is unable to mediate cultural, class and power differences between the patient and provider [36].

### Trust and confidence

Trust and confidence are crucial for obtaining equality in a health care system, as well as for fostering a good patient/provider relationship. Research highlights their potential value in understanding the performance of health care systems [37]. According to the interviewed midwives, mistrust can be established because of health care policies (e.g. X-raying pregnant women from some countries). Although health care workers in Sweden are not required to report immigration law violations [38], miscommunication can arise due to language and cultural barriers and patient circumstances (e.g. inaccurate registration of the children in some Somalian families). The ability to communicate correlates with levels of trust [39]. Miscommunication results in lower levels of trust in health care, a relationship that can cause costly problems for society [40]. Using the concept of public trust in health care, Straten et al. [41] combine consideration of inter-personal, organizational and system trust. The results of this study show that the midwives are working hard on establishing inter-personal trust; they try to act in the patients’ best interests [42]. With regards to patient-caregiver trust, the common issues include the patient focus of the caregiver, caregiver competence and quality of care, communication and co-operation and supportive structures and resources. However, the differences between countries, levels of education and the role of non-western medical traditions [43] might invalidate such an approach. It is well known that minority individuals report lower levels of trust than members of the majority [44,45].

### Potential barriers to the use of health care services

Generally, midwives noted that a woman’s level of education and whether she comes from an urban or a rural area can be more important than cultural norms in determining whether or not she seeks health care. Their assumption about the women’s socioeconomic background and length of stay having an effect upon their health care behavior is in agreement with earlier research [46,47].

Another aspect of the study of health care inequality is to consider the provision of equal treatment and who is responsible for it. Some midwives believed that it was the society’s and the health care services’ responsibility to be able to provide equal treatment to all citizens; other midwives believed that it was an individual’s (the immigrant patient’s) responsibility to know the system, to speak the language and be able to express herself. Rundström [48] states that ideally, from the macro-sociological perspective, it is the staff who should obtain knowledge and so become skilled in the medical-cultural issues. The micro-sociological perspective focuses on the individual responsibility for health or individuals’ ability to learn the rules, norms and behaviors which exist and to adapt to them without feeling their integrity or culture is violated, even if she/he is confronted with something unexpected [49].The results of the interviews show that some midwives believe that the vulnerable groups (immigrants, among others) face more difficulties in getting adequate care. The vulnerable groups suffer because of the structural conditions in the society and health care system and not because of their inability to adapt to health care services. It is important that attempts to identify weaknesses in health care policies do not degenerate into a position that blames the victim. The ideology of individual responsibility for health tends to obscure the reality of the impact of social inequality on health and it views the individual as being essentially independent of his or her surroundings [50].

### Cultural differences and transcultural health care

Cultural background, cultural beliefs and expectations were other contributing factors to inequalities in health care. Different systems of cultural beliefs and practices and different views and expectations may lead to conflicts between immigrant women and their care givers [51,52]. The results of this study show that some midwives have developed an appropriate way to provide information and to offer choices and let the immigrant women feel that they are in control of their own bodies and health care decisions, i.e., to see them as individuals and not as a group. Rice [51] argues that one of the factors that may lead to miscommunication is that immigrant women are not given information and allowed to make their own choices. They should be offered a choice and their individual needs should be considered [53]. As one of the interviewed midwives emphasized *“It is important to let the immigrant woman herself say what she needs and what she wants”*.

The interviewed midwives felt that “health care services should be more aware that people come from different cultures”. Furthermore, they all agreed that having culturally diverse health care staff was important means through which to provide culturally sensitive health care. Previous research shows that receiving culturally appropriate services from health care staff is more than simply a patient’s right; in reality, it is a key factor in the safety and quality of patient care and moves away from a “one size fits all” approach that negatively affects the quality of care for diverse patients [54]. Transcultural values may result in fewer communication problems because of language and cultural differences [55] and the employment of bilingual and bicultural staff, especially in obstetric services, is recommended [33]. The results show that the midwives’ knowledge of the concept of transcultural health care was limited. However, midwives have a professional and culturally sensitive approach, thanks to their long experience and genuine interest in their work. The results also show that there is a need for continuous training in cultural diversity. The interviewed midwives expressed the opinion that there should be more on the subject of transcultural care in their education and training program. Previous research [56-58] recognizes the need for educating health care staff on transcultural health care issues.

Some midwives regarded transcultural health care as *“cultural communication”* and viewed immigrants as a group of “others”^1^ to be studied and analyzed. The danger of the “seeing immigrants as a group” approach is that it assigns everyone to a particular group with the same life experiences and the same cultural behaviors. Maintaining a focus on “others” may reinforce negative qualities and lead to stereotyping and discrimination [59]. Transcultural care is about providing culturally relevant care [57]. It emphasizes the requirement for the development of self-reflection on one’s own cultural identities as an individual and health professional and toward a greater focus on the patient as an individual [56]. It is about cultural awareness and openness [57] or as Campinha-Bacote’s model [60] explains, it is about embodying the following attributes: awareness of one’s own biases and prejudices toward other cultures, knowledge about culture in general, the ability to conduct accurate cultural assessments and interpersonal skills in cross-cultural encounters. Another crucial issue related to transcultural health care that midwives raised in the interviews is the idea of ethnic health care services. The midwives were all negatively disposed to the idea. They believed that ethnic health care services would lead to increased segregation, reinforce prejudices and provide low quality care since patients would get fewer resources under such a system. They also believed that qualified health care staff would not want to work in such services. Kai [61] stressed that most people from diverse ethnic communities do not want ethnic services. Like everyone else they just desire good quality services. If regarding immigrants as a group is a form of ethnocentrism and ethnic discrimination, then providing ethnic health care services would be the other side of the coin, i.e., it would be providing “culturally relativist” health care.

### Methodological considerations

One limitation of this study may be the limited number of interviewees used. However, the number of participants was enough to attain adequate thematic saturation because of sample homogeneity: they were all female, midwives and worked with the same category of patients. Guest et al., [62] stated that the more similar participants in a sample are in their experiences with respect to the research domain the sooner we should expect to reach saturation. Another limitation may be that the results may have suffered from selection bias, i.e., the sampling method may have affected the findings. This may have occurred due to the fact that the study participants were chosen from two municipalities in districts that had a higher number of immigrants. Different results may have been obtained if the study had also included interviews with midwives who work in districts with fewer immigrants. Such a selection might have improved the investigation of the role of immigrant patients’ socioeconomic situation. A well-selected and diversified sample is important. If the findings are based on the range of social settings that is likely to contribute to a particular experience, it strengthens the generalizability of the conclusions [63]. The interview location was planned according to the wishes of the interviewee, as the aim was to create a relaxed setting. The subjectivity of the researcher is another methodological issue that can be discussed. Morse [17] states that in order to conduct valid research it is imperative that the researcher be aware of personal bias or agenda. Research questions may not be value-free but may even reflect the researcher’s values. In this study, the questions about transcultural health care were based on the general discussion on transcultural health care in Sweden and the author’s previous research and knowledge in the field. They could therefore be seen as leading questions.

## Conclusions

Midwives believe that health care inequality among immigrants may be the result of miscommunication which may arise due to a shortage of meeting time, language barriers, different systems of cultural beliefs and practices and limited patient-caregiver trust. Immigrants face more difficulties in seeking health care and in receiving adequate levels of care. The level of education, country of origin and length of stay in Sweden is believed to influence immigrants’ health care seeking behavior. An interesting difference was observed among the midwives’ views; some midwives are sensitive to individual and intra-group differences while other midwives view immigrants as a group of “others”. The findings of the study suggest that more research is needed about the potentials of educating health care staff on the provision of transcultural health care and regarding midwives’ attitudes toward subgroup-specific health care services. This might be a starting point in developing strategies for reducing ethnic inequalities in the health care system.

## Endnotes

Edward Said argues that ‘otherness’ serves to re-impose colonial domination by suggesting that western values, beliefs and forms of culture are imposed to counter the inherently negative ‘traits’ of these so called inferior cultures (Said E.W., Orientalism, New York: Pantheon, 1978).

## Competing interests

The author declared that he has no competing interest.

## Authors’ contributions

SA is the only author of the manuscript and takes full responsibility for the manuscript.

## Authors’ information

Department of Public Health – University of Skövde & School of Health, Care and Social Welfare – University of Mälardalen– Sweden. Sharareh Akhavan is Senior Lecturer in Public Health and working on several research projects related to immigrants’ health.

## References

[B1] The National Board of Health and welfare. The 2009 Swedish Health Care Report 2010 Edita Västra Aros, Västerås http://www.socialstyrelsen.se/publikationer2009/2009-9-18

[B2] The World Health Organization database http://www.euro.who.int/__data/assets/pdf_file/0010/96409/E88669.pdf

[B3] Djurfeltd A Huldt E Immigrants and health care. A right-based utilitarian approach 2007 Department of political science, Lund University

[B4] Statistics Central Board Ohälsa och sjukvård 1980–2005, Levnadsförhållandena 2006 Rapport nr. 113. [Trans: Ill-health and health care between 1980 and 2005, Life conditions]

[B5] Akhavan S The health and working conditions of female immigrants in Sweden PHD thesis 2006 Public Health Department, Karolinska Institute

[B6] ACOG. American College of Obstetricians and Gynecologists Committee on Health Care for Underserved Women ACOG committee opinion: cultural competency in health care Int J Gynecol Obstet 1998 62 96 99 9722136

[B7] The Swedish association of health professionals database SCB-Tryck, Örebro https://www.vardforbundet.se/In-English

[B8] Statistics Central Board Befolkningsstatistik i sammandrag 1960–2005 2005 EO Print, Stockholm [Trans: Population statistics between 1960 and 200]

[B9] Hogstedt C Backhans M Bremberg S Lundgren B Törnell B Wamala S Välfärd, jämlikhet och folkhälsa – vetenskapligt underlag för begrepp, mått och indikationer 2003 Statensfolkhälsoinstitutet, EO Print, No.12. [Trans: Welfare, equality and public health – scientific basis for concept, measurement and indications (The Swedish Public Health Board)]

[B10] de los Reyes P Kamali M Bortom vi och dom - Teoretiska reflektioner om makt, integration och strukturell diskriminering 2005 SOU, Edita Norstedts Tryckeri AB, Stockholm No. 41. [Trans: Beyond we and them - theoretical reflections on power, integration and structural discrimination]

[B11] National Board of Health and welfare Hälso- och sjukvård - lägesrapporter 2007 2008 Stockholm [Trans: Health and health care, Current report for 2007]. http://www.socialstyrelsen.se/Lists/Artikelkatalog/Attachments/8864/2008-131-7_20081317_rev.pdf.

[B12] Social Department Hälso-och sjukvård inför 90-talet, Invandrarna i hälso-och sjukvården, HS 90 1984 SOU, Socialstyrelsen, Stockholm No. 45: [Trans: Health and healthcare in the 90s, Immigrants in health care]

[B13] Fagerli RA Lien ME Wandel M Health worker style and trustworthiness as perceived by Pakistani–born persons with type 2 diabetes in Oslo, Norway Health: An interdisciplinary Journal for the Social Study of Health, Illness and Medicine 2007 11 1 109 129 10.1177/1363459307070810 17158834

[B14] Wamala S Bildt C Adrian G Maina Ahlberg B Svenska empiriska studier av sambandet mellan diskriminering och psykisk och fysisk ohälsa bland utlandsfödda. I Hälsa, vård och strukturell diskriminering. Redaktör: Adrian G, Maina Ahlberg B 2006 SOU, Edita Sverige AB, Stockholm 35 84 No. 78: [Trans: Swedish empirical studies on the relationship between discrimination and psychological and physical ill-health among non-natives]

[B15] National Institute of Public Health Särbehandlad och kränkt 2005 Edita Sverige AB, Stockholm No. 49. [Trans: Discriminated against and insulted]

[B16] Hedemalm A Immigrants with heart failure – a descriptive comparative study of symptoms, self care, social support, care and treatment PhD thesis 2007 The Sahlgrenska Academy, Gothenburg University

[B17] Morse JM Field PA Nursing research – The application of qualitative approaches 1996 2 Chapman & Hall, London

[B18] Svensson PG Starrin B Kvalitativa studier i teori och praktik 1996 Studentlitteratur, Lund [Trans: Qualitative studies in theory and practice]

[B19] Statistics Central Board Integration: en beskrivning av läget I Sverige 2008, Rapport 1 [Trans: Integration: A description of the situation in Sweden]

[B20] Lofland J Lofland LH Typological Systems - Analyzing social settings 1995 3 Wadsworth, Belmont, Cal

[B21] Graneheim UH Lundman B Qualitative content analysis in nursing research: concepts, procedures and measures to achieve trustworthiness Nurse Educ Today 2004 24 2 105 112 10.1016/j.nedt.2003.10.001 14769454

[B22] MacQueen KM McLellan E Kay K Milstein B Codebook development for team-based qualitative research Cultural Anthropology Methods Journal 1998 10 2 31 36

[B23] Miles MB Huberman AM Qualitative Data Analysis 1994 2 Sage Publications, Thousand Oaks, CA

[B24] Ryan GW Bernard HR Denzin NK, Lincoln YS Data management and analysis methods Handbook of qualitative research 2000 2 Sage Publications, Thousand Oaks, CA 769 802

[B25] Hjärn A Haglund B Persson G Rosen M Is there equity in access to health services for ethnic minorities in Sweden? Eur J Public Health 2001 11 147 152 10.1093/eurpub/11.2.147 11420800

[B26] Helman CG Culture, health and illness 1994 3 Butterworth-Heineman, London

[B27] Fortier JP Strobel C Aguilera E Language barriers to health care: federal and state initiatives, 1990–1995 J Health Care Poor Underserved 1998 9 81 100 10.1353/hpu.2010.0692

[B28] Small R Rice PL Yelland J Lumley J Mothers in a new country: the role of culture and communication in Vietnamese, Turkish and Filipino women’s experiences of giving birth in Australia Women Health 1999 28 77 101 10.1300/J013v28n03_06 10374809

[B29] Timmins CL The impact of language barriers on the health care of Latinos in the United States: A review of the literature and guidelines for practice J Midwifery Womens Health 2002 47 2 80 96 10.1016/S1526-9523(02)00218-0 12019990

[B30] Tang SY Interpreter services in healthcare: policy recommendations for healthcare agencies J Nurs Adm 1999 29 23 9 10377922 10.1097/00005110-199906000-00006

[B31] Carrasquillo O Orav EJ Brennan TA Burstin HR Impact of language barriers on patient satisfaction in an emergency department J Gen Intern Med 1999 14 82 87 10.1046/j.1525-1497.1999.00293.x 10051778

[B32] Nguyen TU Tran JH Kagawa-Singer M Foo MA A qualitative assessment of community-based breast health navigation services for Southeast Asian women in Southern California: recommendations for developing a navigator training curriculum Am J Public Health 2011 101 1 87 93 10.2105/AJPH.2009.176743 21088273 PMC3000717

[B33] Caley T Multiculturalism and the midwife Austrian College of Midwives Incorporated Journal 1998 11 2 25 29 10.1016/S1031-170X(98)80031-7 10531814

[B34] Bureau of the Census Census 2000 Supplementary Survey Summary Tables: Table P003: Hispanic or Latino by race-universe: total population http://factfinder.census.gov/servlet/DTTable?_ts_25902237020

[B35] Richardson A Thomas VH Richardson A Reduced to nods and smiles. Experiences of professionals caring for people with cancer from black and ethnic minority groups Eur J Oncol Nurs 2006 10 93 101 10.1016/j.ejon.2005.05.002 16019261

[B36] Kaufert JM Putsch RW Communication through interpreters: ethical dilemmas arising from differences in class, culture, language, and power J Clin Ethics 1997 8 71 87 9130112

[B37] Gilson L Trust and the development of health care as a social institution Soc Sci Med 2003 56 1453 1468 10.1016/S0277-9536(02)00142-9 12614697

[B38] Statens offentliga utredningar Vård efter behov och på lika villkor - en mänsklig rättighet. Betänkande av utredning om vård för papperslösa m fl 2011 SOU, Elanders Sverige AB, Stockholm No. 48. [Trans: Health care according to need and on equal conditions - A human right]

[B39] Franzini L Self-rated health and trust in low-income Mexican-origin individuals in Texas Soc Sci Med 2008 67 1959 1969 10.1016/j.socscimed.2008.09.030 18952340

[B40] Bauer HM Rodriguez MA Szkupinski Quiroga S Flores-Ortiz YG Barriers to health care for abused Latina and Asian immigrant women J Health Care Poor Underserved 2000 11 1 33 44 10.1353/hpu.2010.0590 10778041

[B41] Straten GFM Friele RD Groenewegen PP Public trust in Dutch health care Soc Sci Med 2002 55 227 234 10.1016/S0277-9536(01)00163-0 12144137

[B42] Birungi H Injections and self-help: risk and trust in Ugandan health care Soc Sci Med 1998 47 10 1455 1462 10.1016/S0277-9536(98)00194-4 9823041

[B43] Goudgea J Gilsona L How can trust be investigated? Drawing lessons from past experience Soc Sci Med 2005 61 1439 1451 10.1016/j.socscimed.2004.11.071 16005779

[B44] Alesina A La Ferrara E Who trusts others? J Public Econ 2002 85 207 234 10.1016/S0047-2727(01)00084-6

[B45] Leigh A Trust, inequality and ethnic heterogeneity Econ Rec 2006 82 258 268 280 10.1111/j.1475-4932.2006.00339.x

[B46] Xu KT Broders TF Does being an immigrant make a difference in seeking physician services? J Health Care Poor Underserved 2008 19 2 380 390 10.1353/hpu.0.0001 18469411

[B47] Blackwell DL Martinez ME Gentleman JF Sanmartin C Berthelot JM Socioeconomic status and utilization of health care services in Canada and the United States: findings from a binational health survey Med Care 2009 47 11 1136 1146 10.1097/MLR.0b013e3181adcbe9 19786920

[B48] Rundström B Invandrare I vård och omsorg. En fråga om bemötande av äldre. Rapport till utredning om bemötande av äldre 1997, SOU rapport 76 [Trans: Immigrants in the health care system. The treatment of the elderly. Report submitted to the panel on the treatment of the elderly]

[B49] Goffman E Asylums 1961 Penguin, Essays on the social situation of mental patients and other inmates. London

[B50] Carwford R Mackay L, Soothill K, Melia K You are dangerous to your health: the ideology and politics of victim blaming Classic texts in health care 1998 Butterworth-Heinemann Publishing Ltd, Oxford 84 90

[B51] Rice PL What women say about their childbirth experiences: the case of Hmong women in Australia Journal of reproductive and infant psychology 1999 17 3 237 253 10.1080/02646839908404592

[B52] Barbosa Da Silva A Ljungquist M Vårdetik för ett mångkulturellt Sverige. En teoretisk och empirisk analys av några nödvändiga villkor för en öppen holistisk vård i ett pluraliskt samhälle 2003 Studentlitteratur, Lund [Trans: Ethical care for a multi-cultural Sweden. A theoretical and empirical analysis of some important conditions for an open, holistic health care system in a pluralistic society]

[B53] Maputle MS Jali MN Dealing with diversity: incorporating cultural sensitivity into midwifery practice in the tertiary hospital of Capricorn District, Limpopo Province Curationis 2006 29 4 61 69 17310746

[B54] Wilson-Stronks AL The role of nursing in meeting health care needs of diverse populations J Nurs Care Qual 2008 23 4 289 291 10.1097/01.NCQ.0000336669.79853.38 18806642

[B55] Salt K Step 3: provides culturally competent care - The coalition for improving maternity services J Perinat Educ 2007 16 Suppl 1 23 24 10.1624/105812407X173155 PMC2409131 18523675

[B56] Kai J Beavan J Faull C Dodson L Beighton A Professional uncertainty and disempowerment responding to ethnic diversity in health care: A qualitative study PLoS Med 2007 4 11 1766 1775 10.1371/journal.pmed.0040323 PMC2071935 18001148

[B57] Murphy SC Mapping the literature of transcultural nursing J Med Libr Assoc 2006 94 Suppl 2 143 151 PMC1463039 16710461

[B58] Ryan M Carlton KH Ali N Transcultural nursing concepts and experiences in nursing curricula J Transcult Nurs 2000 11 4 300 307 10.1177/104365960001100408 11982039

[B59] Seaton PL Cultural care in nursing: a critical analysis PhD thesis 2010 University of Technology, Sydney

[B60] Campinha-Bacote J The quest for cultural competence in nursing care Nurs Forum 1995 30 4 19 25 10.1111/j.1744-6198.1995.tb00483.x 8700744

[B61] Kai J Kai J Toward quality in health care for a diverse society Ethncity, health and primary care 2006 2 Oxford University Press, Oxford 27 37

[B62] Guest G Bunce A Johnson L How many interviews are enough? An experiment with data saturation and variability Field Methods 2006 18 1 59 82 10.1177/1525822X05279903

[B63] Daly J Lumley J Bias in qualitative research designs Aust N Z J Public Health 2002 26 4 299 300 10.1111/j.1467-842X.2002.tb00174.x 12233947

